# Racial and Ethnic Disparities in Outcomes Among Newborns with Congenital Diaphragmatic Hernia

**DOI:** 10.1001/jamanetworkopen.2023.10800

**Published:** 2023-04-28

**Authors:** Shelby R. Sferra, Pooja S. Salvi, Annalise B. Penikis, Jennine H. Weller, Joseph K. Canner, Matthew Guo, Abigail J. Engwall-Gill, Daniel S. Rhee, Joseph M. Collaco, Amaris M. Keiser, Daniel G. Solomon, Shaun M. Kunisaki

**Affiliations:** 1 Division of General Pediatric Surgery, Department of Surgery, Johns Hopkins University School of Medicine, Baltimore, Maryland; 2 Division of Pediatric Surgery, Department of Surgery, Yale University School of Medicine, New Haven, Connecticut; 3 Department of Surgery, Yale University School of Medicine, New Haven, Connecticut; 4 Division of Pediatric Pulmonology, Department of Pediatrics, Johns Hopkins Children’s Center, Baltimore, Maryland; 5 Division of Neonatal-Perinatal Medicine, Department of Pediatrics, Johns Hopkins Children’s Center, Baltimore, Maryland

## Abstract

**Importance:**

There is some data to suggest that racial and ethnic minority infants with congenital diaphragmatic hernia (CDH) have poorer clinical outcomes.

**Objective:**

To determine what patient- and institutional-level factors are associated with racial and ethnic differences in CDH mortality.

**Design, Setting, and Participants:**

Multicenter cohort study of 49 US children’s hospitals using the Pediatric Health Information System database from January 1, 2015, to December 31, 2020. Participants were patients with CDH admitted on day of life 0 who underwent surgical repair. Patient race and ethnicity were guardian-reported vs hospital assigned as Black, Hispanic (White or Black), or White. Data were analyzed from August 2021 to March 2022.

**Exposures:**

Patient race and ethnicity: (1) White vs Black and (2) White vs Hispanic; and institutional-level diversity (as defined by the percentage of Black and Hispanic patients with CDH at each hospital): (1) 30% or less, (2) 31% to 40%, and (3) more than 40%.

**Main Outcomes and Measures:**

The primary outcomes were in-hospital and 60-day mortality. The study hypothesized that hospitals managing a more racially and ethnically diverse population of patients with CDH would be associated with lower mortality among Black and Hispanic infants.

**Results:**

Among 1565 infants, 188 (12%), 306 (20%), and 1071 (68%) were Black, Hispanic, and White, respectively. Compared with White infants, Black infants had significantly lower gestational ages (mean [SD], White: 37.6 [2] weeks vs Black: 36.6 [3] weeks; difference, 1 week; 95% CI for difference, 0.6-1.4; *P* < .001), lower birthweights (White: 3.0 [1.0] kg vs Black: 2.7 [1.0] kg; difference, 0.3 kg; 95% CI for difference, 0.2-0.4; *P* < .001), and higher extracorporeal life support use (White: 316 patients [30%] vs Black: 69 patients [37%]; χ^2^_1_ = 3.9; *P* = .05). Black infants had higher 60-day (White: 99 patients [9%] vs Black: 29 patients [15%]; χ^2^_1_ = 6.7; *P* = .01) and in-hospital (White: 133 patients [12%] vs Black: 40 patients [21%]; χ^2^_1_ = 10.6; *P* = .001) mortality . There were no mortality differences in Hispanic patients compared with White patients. On regression analyses, institutional diversity of 31% to 40% in Black patients (hazard ratio [HR], 0.17; 95% CI, 0.04-0.78; *P* = .02) and diversity greater than 40% in Hispanic patients (HR, 0.37; 95% CI, 0.15-0.89; *P* = .03) were associated with lower mortality without altering outcomes in White patients.

**Conclusions and Relevance:**

In this cohort study of 1565 who underwent surgical repair patients with CDH, Black infants had higher 60-day and in-hospital mortality after adjusting for disease severity. Hospitals treating a more racially and ethnically diverse patient population were associated with lower mortality in Black and Hispanic patients.

## Key Points

**Question:**

What factors are associated with mortality differences among newborns with congenital diaphragmatic hernia (CDH) from different racial and ethnic groups?

**Findings:**

In this multicenter cohort study of 1565 patients, Black infants had significantly higher in-hospital mortality compared with other racial and ethnic groups. Hospitals in more racially and ethnically diverse communities were associated with lower 60-day mortality among Black and Hispanic infants, without affecting mortality in White infants.

**Meaning:**

Despite ongoing health outcome disparities in Black infants with CDH, these findings suggest evidence of improved outcomes observed in Black and Hispanic patients managed at hospitals caring for larger racial and ethnic minority patient populations.

## Teaser

This multicenter cohort study evaluates racial and ethnic disparities in outcomes among newborns with congenital diaphragmatic hernia.

## Introduction

There is a growing body of evidence that morbidity and mortality are heightened in racial and ethnic minority populations in the US, a discrepancy widely attributed to the effect of health care disparities.^1,2,3,4,5,6,7,8,9,10^ This is most apparent in the Black and Hispanic communities, affecting not only adults but also infants and young children.^1,2,3,4,5,6,7,8,9,10,11,12,13,14,15,16^ Black mothers, for example, are more likely to deliver at hospitals with increased rates of complications compared with White mothers.^17^ The discrepancy is further magnified as Black neonates are more likely to be born preterm with low to very low birth weights, increasing their predisposition to immediate complications.^18^ After birth, Black infants have been shown to receive care in inferior neonatal intensive care units due to insurance status and financial and racial polarization.^19,20,21,22^ These disparities may be amplified in neonates with a congenital disease diagnosis, with disparate care affecting both prenatal diagnosis as well as postnatal resuscitation and definitive care strategies.

Congenital diaphragmatic hernia (CDH) is one of the most important congenital anomalies managed by pediatric surgeons according to incidence, resource utilization, and cost.^23^ Failed embryonic closure of the diaphragm in affected fetuses results in intrathoracic abdominal viscera herniation and varying degrees of pulmonary hypoplasia, pulmonary hypertension, and cardiac dysfunction at birth.^24,25,26^ Prenatally diagnosed cases require increased surveillance and, in the most severe cases, fetal endoscopic tracheal occlusion has been shown to improve survival with acceptable long-term morbidity.^27,28^ In the majority of patients, survival is improved with delivery at high-volume, designated CDH centers that have a multidisciplinary care approach and access to adjuvant therapies, including extracorporeal life support (ECLS).^29,30^ Despite such intensive care, the overall neonatal mortality rate in CDH approaches 25% to 30%.^24,25^ The mortality rate in CDH may be even higher in racial and ethnic minority patient populations, but these findings are based on data from nearly 20 years ago.^31,32,33^ Moreover, the impact of disease severity, socioeconomic status (SES), and institution-specific factors on outcome disparities in infants with CDH has not been well elucidated.^31,32,33^

The primary aim of our study was to evaluate mortality disparities in CDH using a large, contemporary national database. A secondary aim was to determine how institution-specific factors might correlate with clinical outcomes among different racial and ethnic cohorts with CDH. Our group speculated that hospitals serving larger racial and ethnic minority communities might be associated with a survival advantage among Black and Hispanic infants.

## Methods

### Study Design, Setting, Participants

This was a retrospective, multicenter cohort study using demographic, clinical, and outcome data from the Children’s Hospital Association Pediatric Health Information System (PHIS). The PHIS database contains administrative and billing data from 49 children’s hospitals in the US.^34^ Data quality and reliability are assured by the Children’s Hospital Association, participating institutions, and Truven Health Analytics.^35,36^ The *International Classification of Diseases, Ninth Revision (ICD-9)* and *International Statistical Classification of Diseases and Related Health Problems, Tenth Revision (ICD-10)* diagnosis and procedure codes were used to identify patient-level data.

Institutional review board approval was obtained at Johns Hopkins University and exemption of patient consent was granted. Review board approval was deemed exempt at Yale University because the data was publicly available and deidentified. The PHIS database was queried for patients with CDH using *ICD-9* and *ICD-10* diagnoses codes between January 1, 2015, and December 31, 2020. Patients were included if they were admitted on day of life 0 and underwent surgical repair of their diaphragmatic hernia (*ICD-9* and *ICD-10* diagnosis and procedure codes, eTable in Supplement 1). Patients who did not undergo surgical repair and those with missing race and ethnicity data were excluded from analysis. This study followed the Strengthening the Reporting of Observational Studies in Epidemiology (STROBE) reporting guideline for cohort studies.^37^

### Variables

Data collection included demographic variables, markers of SES, postnatal markers of disease severity, hospital clinical course, and in-hospital outcomes. Household income was measured by median income quartile as determined by the zip code of residence linked to US Census Bureau data.^38^ Cardiovascular anomalies were defined as heart and great vessel malformations, endocardium diseases, cardiomyopathies, conduction disorders and dysrhythmias, cardiac devices, and/or transplantation.^39^ Hospital case volume was defined by the mean number of CDH repairs per center from 2015 to 2020. Low case volume was less than 10 cases per year, whereas high case volume was 10 or more cases per year according to prior work.^29,40,41^

Race and ethnicity reporting in the PHIS database is coded by administrative staff according to hospital-specific guidelines, including patient/guardian self-report or hospital registration assignment.^42^ Race categories were American Indian or Alaskan Native, Asian, Black or African American, Native Hawaiian or Pacific Islander, White, or other (other included multiracial and unknown). Ethnicity categories were Hispanic/Latino or not Hispanic/not Latino. The racial and ethnic groups that were used in the analysis included Black (Black, non-Hispanic), Hispanic (White or Black Hispanic), and White (White, non-Hispanic). American Indian or Alaskan Native, Asian, Native Hawaiian or Pacific Islander, or other racial and ethnic groups were excluded due to the relatively low number and thus inability to deduce meaningful or valid results. At each institution, we also quantified institutional-level diversity, defined as the percentage of Black and Hispanic patients with CDH at each hospital (as determined by *ICD-9* and *ICD-10* diagnosis code; eTable in Supplement 1). Racial and ethnic diversity levels of (1) 30% or less (2) 31%-40%, and (3) greater than 40% were established according to the 2020 US Census data, in which 71% of the US population self-identified as White.^43^

The primary outcomes of interest were 60-day and in-hospital mortality rates. Three markers of disease morbidity, namely hospital length of stay, discharge to home, and tracheostomy, were used as secondary outcome measures.

### Statistical Analysis

A *t* test was performed for parametric data, whereas a Wilcoxon rank sum was performed for nonparametric data. Continuous data were presented as mean (SD) vs median (IQR). A Pearson χ^2^ test was used to analyze categorical variables. The majority patient population served as the reference group for each comparison.

Sixty-day mortality trends of Black, Hispanic, and White patients according to institutional-level racial and ethnic diversity were assessed with a Kaplan-Meier estimate and significance was determined with a Wilcoxon (Breslow) test. To account for loss-to-follow-up, a Cox proportional regression analysis was used to compare the outcome of multiple variables on the 60-day risk of mortality. A clustered sandwich estimation by hospital was used to allow for intragroup correlation.^44^ To reduce the effect of immortal time bias, the person-time variable (day 0) started on the day of surgery. Variables with a known effect on CDH mortality were used in the Cox regression. Downstream effectors and causal intermediates of race were excluded from analyses.^45^ Statistical analyses were performed with Stata version 16.1 (StataCorp). Significance was defined as a 2-sided *P*≤.05. Data were analyzed from August 2021 to March 2022.

## Results

### Baseline Characteristics

There were 1893 patients with CDH who underwent surgical repair. There were 328 (17%) patients excluded according to identification as American Indian or Alaskan Native (17 patients), Asian (60 patients), Native Hawaiian or Pacific Islander (7 patients), or other (244 patients). Among the remaining 1565 patients, 188 (12%) were Black, 306 (20%) were Hispanic, and 1071 (68%) were White (Table 1). There was no difference in sex across racial and ethnic groups. The median (IQR) household income of White patients ($57 618 [$45 625-$74 694]) was significantly greater than the income of Black patients ($44 375 [$32 186-$57 207]; *z *score, 9.0; *P* < .001) and Hispanic patients ($50 125 [$40 909-$64 332]; *P* < .001). The White patient population used significantly higher rates of commercial insurance (634 [59%]) compared with Black (27 [14%]; χ^2^_1_ = 128.9; *P* < .001) and Hispanic patients (72 [24%]; χ^2^_1_ = 121.2; *P* < .001).

**Table 1.** Baseline Characteristics of Infants With Congenital Diaphragmatic Hernia, by Race and Ethnicity

Characteristic	Overall (n = 1565)	White (n = 1071) (reference)	Black (n = 188)	*P* value	Hispanic (n = 306)	*P* value
Demographics						
Sex, No. (%)						
Male	937 (60)	627 (59)	117 (62)	.35	193 (63)	.16
Female	628 (40)	444 (41)	71 (38)		113 (37)	
Household income, median (IQR), US $^a^	54 278 (43 048-70 152)	57 618 (45 625-74 694)	44 375 (32 186-57 207)	<.001	50 125 (40 909-64 332)	<.001
Payer status, No. (%)						
Commercial	733 (47)	634 (59)	27 (14)	<.001	72 (24)	<.001
Medicaid	733 (47)	372 (35)	155 (83)		206 (67)	
Uninsured	99 (6)	65 (6)	6 (3)		28 (9)	
Disease severity						
Gestational age, mean (SD), wk	37.5 (2)	37.6 (2)	36.6 (3)	<.001	37.8 (2)	.09
Birthweight, mean (SD), kg	3.0 (1)	3.0 (1)	2.7 (1)	<.001	3.1 (1)	.15
Apgar score at 1 min, mean (SD)	4.6 (3)	4.6 (3)	3.7 (3)	<.001	4.8 (3)	.33
Apgar score at 5 min, mean (SD)	6.4 (3)	6.5 (3)	5.6 (3)	<.001	6.7 (2)	.24
Cardiovascular anomalies, No. (%)^b^	759 (48)	506 (47)	101 (54)	.10	152 (50)	.45
Institutional						
Urban center, No. (%)	1259 (80)	822 (77)	176 (94)	<.001	261 (85)	<.001
Volume, No. (%)						
Low volume	884 (56)	567 (53)	107 (57)	.31	210 (69)	<.001
High volume	681 (44)	504 (47)	81 (43)		96 (31)	
Racial and ethnic diversity, No. (%)^c^						
≤30%	465 (30)	393 (37)	51 (27)	<.001	21 (7)	<.001
31%-40%	427 (27)	334 (31)	41 (22)		52 (17)	
>40%	673 (43)	344 (32)	96 (51)		233 (76)	

^a^ Median household income was estimated by zip code.

^b^ Cardiovascular anomalies include heart and great vessel malformations, endocardium diseases, cardiomyopathies, conduction disorders and dysrhythmias, cardiac devices, and/or transplantation.

^c^ Racial and ethnic diversity is the percentage of Black and Hispanic patients with congenital diaphragmatic hernia at each institution.

White infants, compared with Black infants, were more likely to be full term (mean [SD] gestational age, White: 37.6 [2] weeks vs Black: 36.6 [3] weeks; difference, 1.0 week; 95% CI for difference, 0.6-1.4; *P* < .001), have a higher mean (SD) weight (White: 3.0 [1.0] kg vs Black: 2.7 [1.0] kg; difference, 0.3 kg; 95% CI for difference, 0.2-0.4; *P* < .001), and have higher mean (SD) Apgar scores at 5 minutes (White: 6.5 [3] vs Black: 5.6 [3]; difference, 0.9; 95% CI for difference, 0.4-1.3; *P* < .001). White patients were treated at high-volume centers at a comparable rate to Black patients, but more often than Hispanic (White: 504 [47%] vs Hispanic 96 [31%]; χ^2^_1_ = 23.8; *P* < .001) patients. White patients were treated at hospitals with institutional-level racial and ethnic diversity of 30% or less at higher rates compared with other groups (White: 393 [37%] vs Black: 51 [27%]; χ^2^_1_ = 25.3; *P* < .001 vs Hispanic 21 [7%]; χ^2^_1_ = 197.6; *P* < .001).

### Hospital Course

Infants with CDH from all racial and ethnic categories were admitted to the ICU for a comparable number of days (Table 2). White patients required mechanical ventilation for significantly less time than Black patients (White: mean [SD] 41 [71] days vs Black: 64 [101] days; difference, −22 days; 95% CI for difference −34 to −10; *P* < .001). White patients also used less inhaled nitric oxide (White: mean [SD] 14 [28] days vs Black: 18 [37] days; difference, −5 days; 95% CI for difference −9 to −.2; *P* = .04). There were no differences in ventilator duration and pulmonary hypertension medication use in Hispanic patients compared with White patients.

**Table 2.** Hospital Course of Infants With Congenital Diaphragmatic Hernia, by Race and Ethnicity

Characteristic	Overall (N = 1565), mean (SD)	White (n = 1071) (reference), mean (SD)	Black (n = 188), mean (SD)	*P* value	Hispanic (n = 306), mean (SD)	*P* value
Time in ICU, d	65 (250)	65 (299)	72 (75)	.77	60 (74)	.77
Time on mechanical ventilation, d	44 (78)	41 (71)	64 (101)	<.001	39 (84)	.63
Time on sildenafil, d	18 (55)	18 (49)	26 (77)	.05	15 (57)	.35
Time on iNO, d	14 (29)	14 (28)	18 (37)	.04	12 (30)	.40
ECLS, No. (%)	457 (29)	316 (30)	69 (37)	.05	72 (24)	.04
ECLS before surgery, No. (%)^a^	195 (43)	137 (43)	26 (38)	.65	32 (44)	.79
Repair while receiving ECLS, No. (%)^b^	126 (65)	86 (63)	18 (69)	.34	22 (69)	.77
Repair after ECLS, No. (%)^b^	69 (35)	51 (37)	8 (31)	.97	10 (31)	.24
ECLS cannulation day of life, d	3 (13)	3 (13)	4 (14)	.40	4 (12)	.53
ECLS decannulation day of life, d	19 (17)	19 (18)	23 (18)	.10	17 (13)	.35
Age at repair, d	8 (20)	7 (16)	13 (38)	<.001	7 (17)	.88

Abbreviations: iNO, inhaled nitric oxide; ECLS, extracorporeal life support; ICU, intensive care unit.

^a^ Denominator reflective of No. patients receiving ECLS.

^b^ Denominator reflective of No. patients receiving ECLS before surgery.

White infants were supported on ECLS at significantly lower rates than Black infants (White: 316 patients [30%] vs Black: 69 patients [37%]; χ^2^_1_ = 3.9; *P* = .05) but at significantly higher rates compared with Hispanic infants (Hispanic: 72 patients [24%]; χ^2^_1_ = 4.2; *P* = .04). Of those placed on ECLS, there were no differences between the racial and ethnic groups in terms of ECLS duration or the timing of their repair in relation to their ECLS status, with most patients being repaired on ECLS (126 patients [64%]).

### Mortality and Morbidity

Table 3 shows mortality and morbidity data. The 60-day mortality rates (White: 99 patients [9%] vs Black: 29 patients [15%]; χ^2^_1_ = 6.7; *P* = .01) and in-hospital mortality rates (White: 133 patients [12%] vs Black: 40 patients [21%]; χ^2^_1_ = 10.6; *P* = .001) were significantly lower in White patients compared with Black patients. There were no mortality differences between White and Hispanic patients. Among CDH survivors, White patients were hospitalized for a significantly shorter time than Black patients (median [IQR], White: 46 [26-83] days vs Black: 55 [33-116] days; *z* score, −3.6; *P* < .001). In addition, White patients were more likely to be discharged home (White: 779 patients [73%] vs Black: 117 patients [62%]; χ^2^_1_ = 8.6; *P* = .003) and were less likely to require a tracheostomy (White: 59 patients [6%] vs Black: 19 patients [10%]; χ^2^_1_ = 5.8; *P* = .02). There were no significant differences in discharge outcomes when comparing White patients to Hispanic patients.

**Table 3.** Hospital Mortality and Morbidity Outcomes in Patients With Congenital Diaphragmatic Hernia, by Race and Ethnicity

Outcome	Overall (N = 1565), No. (%)	White (n = 1071), No. (%) (reference)	Black (n = 188), No. (%)	*P* value	Hispanic (n = 306), No. (%)	*P* value
60-d mortality	145 (9)	99 (9)	29 (15)	.01	30 (10)	.77
In-hospital mortality	211 (14)	133 (12)	40 (21)	.001	38 (12)	>.99
Length of stay, median (IQR), days	47 (27-87)	46 (26-83)	55 (33-116)	<.001	47 (23-89)	.20
Discharge to home	1133 (72)	779 (73)	117 (62)	.003	237 (78)	.10
Tracheostomy at discharge	96 (6)	59 (6)	19 (10)	.02	18 (6)	.80
Gastrostomy at discharge	384 (25)	255 (24)	56 (30)	.08	73 (24)	.99

Kaplan-Meier mortality curves revealed significant differences in 60-day mortality in Black, Hispanic, and White patients according to the racial and ethnic diversity of the infants with CDH treated (Figure). In Black and White patients, racial and ethnic diversity of 31% to 40% was associated with lower mortality. In Hispanic patients, racial and ethnic diversity greater than 40% led to improved mortality rates. In all 3 cohorts, racial and ethnic diversity of 30% or less was associated with higher mortality.

**Figure.**
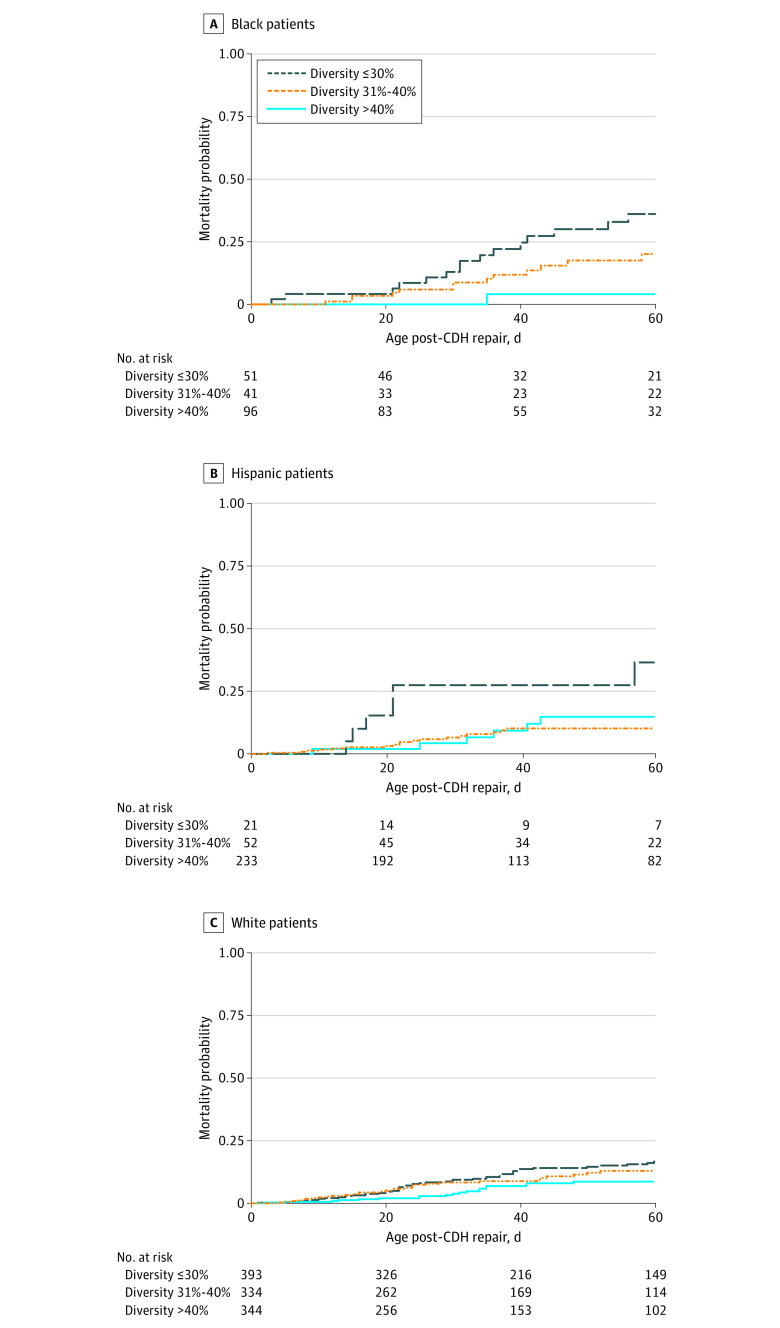
Kaplan-Meier Survival Estimates of 60-Day Mortality in Congenital Diaphragmatic Hernia (CDH) Stratified by Racial and Ethnic Diversity

Cox proportional regression analyses were performed to assess 60-day mortality in Black, Hispanic, and White infants with CDH (Table 4). In all racial and ethnic subgroups, ECLS use was significantly associated with mortality (Black: hazard ratio [HR] 4.66; 95% CI, 1.45-15.01; *P* = .01; Hispanic: HR, 14.82; 95% CI, 4.63-47.42; *P* < .001; White: HR, 7.50; 95% CI, 4.77-11.81; *P* < .001). In Black infants, racial and ethnic diversity of 31% to 40% was associated with lower mortality (HR, 0.17; 95% CI, 0.04-0.78; *P* = .02). In Hispanic patients, racial and ethnic diversity greater than 40% was associated with lower mortality (HR, 0.37; 95% CI, 0.15-0.89; *P* = .03). There was no significant association between racial and ethnic diversity and mortality in White patients.

**Table 4.** Cox Regression Analysis Assessing 60-Day Mortality Factors Associated With Risk in Congenital Diaphragmatic Hernia

Factor	Black		Hispanic		White	
	Hazard ratio (95% CI)	*P* value	Hazard ratio (95% CI)	*P* value	Hazard ratio (95% CI)	*P* value
Disease severity						
Cardiovascular anomalies	1.51 (0.62-3.69)	.37	1.57 (0.81-3.05)	.18	0.76 (0.56-1.04)	.08
Days on nitric oxide	0.98 (0.97-0.99)	.03	0.99 (0.97-1.01)	.30	0.99 (0.98-1.00)	.17
ECLS	4.66 (1.45-15.01)	.01	14.82 (4.63-47.42)	<.001	7.50 (4.77-11.81)	<.001
Institutional						
High volume hospital^a^	0.91 (0.46-1.83)	.80	0.76 (0.41-1.39)	.37	0.74 (0.29-1.86)	.52
Racial and ethnic diversity^b^						
<30%	1 [Reference]	NA	1 [Reference]	NA	1 [Reference]	NA
31%-40%	0.17 (0.04-0.78)	.02	0.40 (0.14-1.12)	.08	0.68 (0.30-1.58)	.38
>40%	0.64 (0.29-1.41)	.27	0.37 (0.15-0.89)	.03	1.15 (0.51-2.63)	.73

Abbreviations: ECLS, extracorporeal life support; NA, not applicable.

^a^ High volume hospitals indicates those with 10 or more cases per year.

^b^ Racial and ethnic diversity is the percentage of Black/Hispanic patients with congenital diaphragmatic hernia treated.

## Discussion

Using a large, national database of 1565 infants across 49 major children’s hospitals, this cohort study sought to evaluate contemporary racial and ethnic mortality differences in CDH. Although we have learned a tremendous amount about disease risk stratification, racial and ethnic outcome disparities have been a relatively unexplored area of CDH clinical research.^25^ Our results revealed that Black infants had significantly higher 60-day and in-hospital mortality rates compared with White infants. Hispanic patients, however, had comparable mortality rates with White patients.

Our data also found that Black patients had evidence of more severe disease according to birth weight, gestational age, and other early postnatal markers of disease. As has been shown in the general population, Black neonates are born at earlier gestational ages compared with White neonates, with a resultant decrease in birthweight. These data are comparable with numerous studies demonstrating discrepancies at birth in Black neonates.^17,18^ There is a growing body of evidence that low SES, early maternal age, lack of prenatal care, and potential racially related stressors are associated with the observed shortened gestation and low birthweight.^18^ In our study, Black patients also required more intensive care treatment, receiving ECLS at a higher frequency and requiring longer courses of pulmonary antihypertensives. Evidence of increased disease severity in Black patients has been suggested in other pediatric conditions as well, specifically in congenital cardiac disease and bronchopulmonary dysplasia.^46,47,48^

The Kaplan-Meier mortality curves in our study confirmed our hypothesis, demonstrating that Black and Hispanic infants had lower mortality rates when treated in hospitals that manage a larger percentage of Black and Hispanic patients. This is starkly contrasted to a diversity of 30% or less, which was associated with significantly higher unadjusted mortality in Black, Hispanic, and White patients. Even after controlling for markers of disease severity, and institutional descriptors such as hospital volume, hospitals with racial and ethnic diversity of 31% to 40% and more than 40% were correlated with lower mortality in Black and Hispanic patients, respectively.

Although our data showed that low levels of patient diversity were negatively associated with outcomes in Black and Hispanic patients, the reasons for this observation remain unknown and are beyond the scope of our study. Nevertheless, we have no evidence there is biologic plausibility for the disparities seen and rather speculate that both unmeasured social determinants of health (SDoH) and clinician bias might be key driving forces for these outcome disparities.^49^ SDoH consist of nonmedical factors that affect an individuals’ health outcomes.^50^ Broadly, the areas involved include education, health care, neighborhood and environment, social and community factors, and economic stability.^50^ Race and ethnicity are inherently intertwined in SDoH and play a key role in the disparities seen in numerous neonatal and pediatric diseases.^1,2,3,4,5,6,7,8,9,10,51,52^ Black race, for example, has been shown to be associated with lower household income and increased socioeconomic deprivation.^53^ This may affect a mother’s ability to access adequate prenatal care due to financial strain and transportation challenges, ultimately predisposing her to preterm labor and infant mortality and morbidity.^54,55^ In those cases with congenital disease, these challenges are magnified as the continued care of the infant requires increased time and resources. Overall, individual and community level socioeconomic and environmental factors add additional levels of acute and chronic stress and increase the complexity in obtaining adequate levels of care compared with those patients living in a more stable environment.^56,57^ These patients are further marginalized at institutions where staff are not accustomed to providing family and patient centered care.^19^ Systemic racism, implicit bias, and societal constructs increase the likelihood that clinicians mislabel patients as unreliable or difficult, rather than addressing the source of their presumed inattention to care.^19^ These factors have been recognized on a national level, as racism and social injustice were named the Determinants of Child Health as the American Pediatric Society Issue of the Year in 2020.^58^ It is clear that social determinants of health predispose Black patients to poor outcomes in CDH and discrimination might further propagate these negative results.

Just as low patient diversity worsened outcomes in racial and ethnic minority populations, higher levels of racial and ethnic diversity were associated with lower mortality in Black and Hispanic patients. This outcome could be due, in part, to increased levels of clinician racial and ethnic diversity at the treating institution. Although our study does not have data on the racial and ethnic diversity of the clinicians at each institution, it is reasonable to hypothesize that clinician racial and ethnic diversity was increased in those hospitals with a more diverse patient population.^59,60^ Both the adult and pediatric literature have shown that racial and ethnic concordance between Black and Hispanic patients and clinicians significantly improves survival.^61,62,63^ This correlation is largely attributed to enhanced trust in the care team and improved communication, leading to better health care utilization.^61,62,63,64^ Furthermore, prior literature has noted that Black clinicians may be more aware of the socioeconomic intricacies and challenges faced by Black patients and are thus better equipped to treat the complexities of care that arise.^62^ However, it is important to recognize that as levels of racial and ethnic diversity are further increased, there is a potential for decreased resources and increased workforce shortages in hospitals that treat primarily racial and ethnic minority patients.^17^ Further studies focusing on hospitals that treat primarily racial and ethnic minority patients should be pursued to evaluate the effects of race and ethnicity in conjunction with resource allocation.

### Limitations

Although our data provide results on a large number of patients with CDH, there are several limitations that deserve mention. First, as with any administrative database study, there were fundamental data collection challenges, such as miscoding and missing data. Additionally, data inconsistencies may be evident such as those seen in household income, whereby heterogeneity may be masked within each zip code. Furthermore, there may be confounding variables that are relevant to assess CDH disease severity but were unavailable in PHIS, including prenatal lung size, liver herniation, intraoperative anomaly size, and additional markers of SES including education level, occupation, deprivation status, distance to specialized care, and public assistance. A second major limitation of our study is that race and ethnicity were regarded as categorical variables which can lead to inherent inaccuracies within the data. Infants from multiracial backgrounds and the obvious heterogeneity within Hispanic communities according to country of origin, language, and immigration status are notable examples. Third, our study included only those patients with CDH that underwent surgical repair. We did not analyze the small fraction (approximately 10%-15%) of patients with CDH where care was withdrawn shortly after birth or who were too ill to tolerate surgery.^65^ This experimental design may explain our slightly lower overall mortality rates. Whether disparities exist among the cohort that did not undergo repair should be a focus of further study. Additionally, our study was unable to analyze the mortality rates and subsequent effects of institutional-level racial and ethnic diversity in additional racial and ethnic minority patients, namely American Indian or Alaskan Native, Asian, or Native Hawaiian or Pacific Islander. Due to small numbers, the results of these analyses were less likely to provide meaningful results. It would therefore be important for our findings to be validated using a larger consortium of patients with CDH .

## Conclusions

This large administrative database study of infants with CDH found that Black patients had significantly higher mortality rates compared with their White counterparts. Our data revealed that hospitals in more racially and ethnically diverse communities were associated with lower 60-day mortality among Black and Hispanic infants, without altering mortality among White infants. To our knowledge, this is the first study of its kind to assess CDH mortality according to diversity of the patient population treated. We believe these data highlight ongoing disparities seen in the care of children with CDH and represent a call to action to better understand how each of us might improve CDH care to infants from all racial and ethnic groups.

## Notes

Supplement 1. **eTable. **International Classification of Diseases (ICD), Ninth and Tenth Revision, Diagnosis and Procedure Codes Used to Acquire Patient Level Data in PHIS Database

Supplement 2.
Data Sharing Statement
