# Supplementary material for: Racial and Ethnic Disparities in Outcomes Among Newborns with Congenital Diaphragmatic Hernia
Source: JAMA Netw Open. 2023 Apr 28;6(4):e2310800. doi: 10.1001/jamanetworkopen.2023.10800 (PMC10148194; doi:10.1001/jamanetworkopen.2023.10800)
Supplement: Supplement 1. — eTable. International Classification of Diseases (ICD), Ninth and Tenth Revision, Diagnosis and Procedure Codes Used to Acquire Patient Level Data in PHIS Database [file jamanetwopen-e2310800-s001.pdf]

Supplemental Online Content

Sferra SR, Salvi PS, Penikis AB, et al. Racial and ethnic disparities in outcomes among newborns with congenital diaphragmatic hernia. *JAMA Netw Open*. 2023;6(4):e2310800. doi:10.1001/jamanetworkopen.2023.10800

eTable. International Classification of Diseases (ICD), Ninth and Tenth Revision, Diagnosis and Procedure Codes Used to Acquire Patient Level Data in PHIS Database

This supplemental material has been provided by the authors to give readers additional information about their work.

© 2023 Sferra SR et al. *JAMA Network Open.*

eTable. *International Classification of Diseases* (*ICD*), Ninth and Tenth Revision, Diagnosis and Procedure Codes Used to Acquire Patient Level Data in PHIS Database

	ICD-9/10 codes
Diagnosis codes for CDH	*ICD*-9-CM: 756.6<br>*ICD*-10-CM: Q79.0/Q79.1
Procedure codes for CDH repair	*ICD*-9/10-PCS: 0BQT0ZZ;0BQT3ZZ; 0BQT4ZZ;0BQR0ZZ; 0BQR3ZZ;0BUR4JZ; 0BQR4ZZ;0BUR4KZ; 0BUR0JZ; 0BQS0ZZ; 0BQS4ZZ; 0BQS3ZZ; 0BUS0JZ; 0BRT07Z; 0BRT0JZ; 0BRT0KZ; 0BRT47Z; 0BRT4JZ; 0BRT4KZ; 0BUT4JZ; 0BUT0JZ; 53.71; 53.72; 53.75; 53.80; 53.83; 53.84

CDH = Congenital Diaphragmatic Hernia

© 2023 Sferra SR et al. *JAMA Network Open.*
