# Supplementary material for: Racial and Ethnic Disparities in Outcomes Among Newborns with Congenital Diaphragmatic Hernia
Source: JAMA Netw Open. 2023 Apr 28;6(4):e2310800. doi: 10.1001/jamanetworkopen.2023.10800 (PMC10148194; doi:10.1001/jamanetworkopen.2023.10800)
Supplement: Supplement 2. — Data Sharing Statement [file jamanetwopen-e2310800-s002.pdf]

Data Sharing Statement

Sferra. Racial and Ethnic Disparities in Outcomes Among Newborns with Congenital Diaphragmatic Hernia. *JAMA Netw Open*. Published April 28, 2023. doi:10.1001/jamanetworkopen.2023.10800

Data
Data available: No
